# Assessing the Potential of *Tortistilus* (Hemiptera: Membracidae) from Northern California Vineyards as Vector Candidates of Grapevine Red Blotch Virus

**DOI:** 10.3390/insects15090664

**Published:** 2024-08-31

**Authors:** Victoria J. Hoyle, Elliot J. McGinnity Schneider, Heather L. McLane, Anna O. Wunsch, Hannah G. Fendell-Hummel, Monica L. Cooper, Marc F. Fuchs

**Affiliations:** 1 School of Integrative Plant Science, Plant Pathology and Plant-Microbe Biology, Cornell University, Geneva, NY 14456, USA; ejm372@cornell.edu (E.J.M.S.); hlm9@cornell.edu (H.L.M.); aw838@cornell.edu (A.O.W.); mf13@cornell.edu (M.F.F.); 2 University of California Cooperative Extension, Napa, CA 94559, USA; hgfendellhummel@ucanr.edu (H.G.F.-H.);

**Keywords:** *Tortistilus wickhami*, grapevine red blotch virus, ITS2, mt-COI, vector candidate, acquisition

## Abstract

**Simple Summary:**

*Tortistilus* and *Spissistilus* are two genera of cryptic treehoppers from the Ceresini tribe. *S. festinus* is a vector of grapevine red blotch virus (GRBV) in vineyards in northern California, USA; however, the transmission capabilities of *Tortistilus* spp. are unknown. In this study, we determined the species of *Tortistilus* found in vineyards in Napa Valley, California, as *T. wickhami*, and determined that only a few specimens but no dissected heads with salivary glands tested positive for GRBV in PCR and qPCR. These results suggested that *T. wickhami* is an unlikely vector of GRBV in northern California vineyards.

**Abstract:**

Ceresini treehoppers are present in northern California vineyard ecosystems, including the closely related *Spissistilus* and *Tortistilus* (Hemiptera: Membracidae). These membracids are not direct pests of wine grapes, but *S. festinus* is a vector of grapevine red blotch virus (GRBV). No information is available on the ability of *Tortistilus* spp. to transmit GRBV. In this study, *Tortistilus* were collected on yellow panel cards across 102 vineyard sites and surrounding areas in Napa Valley, California, USA in 2021–2023. Specimens were morphotyped, sexed and tested for GRBV ingestion and acquisition by multiplex PCR or qPCR. Phylogenetic analysis of the partial sequence of mt-COI and ITS gene fragments of a subset of 40 *Tortistilus* specimens revealed clustering in a monophyletic clade with *T. wickhami* with the former barcode sequence. Only 6% (48/758) of the *T. wickhami* tested positive for GRBV, but none of the heads with salivary glands (0%, 0/50) of the dissected specimens tested positive for GRBV, indicating no virus acquisition. In contrast, half of the dissected heads with salivary glands of *S. festinus* (52%, 12/23), from the same collection vineyard sites, tested positive for GRBV. Together, our findings confirmed the presence of *T. wickhami* in northern California vineyards and suggested a dubious role of this treehopper as a vector of GRBV.

## 1. Introduction

*Spissistilus festinus* (Say) (Hemiptera: Membracidae), the three-cornered alfalfa hopper, is a vector of grapevine red blotch virus (GRBV, genus *Grablovirus*, family *Geminiviridae*) [1,2,3], the causal agent of red blotch disease of grapevines [4]. This disease was first described about 15 years ago and is widely distributed in North America vineyards [5]. Red blotch is a serious concern for grape growers due to its delays in fruit ripening, impact on fruit quality such as lowered anthocyanin and sugar content, and ability to alter the composition and sensory attributes of wines [5,6]. Estimated economic impacts of red blotch disease range from USD 2213 to USD 68,548 per hectare over a 25-year lifespan for a ‘Cabernet Sauvignon’ vineyard in northern California [7].

*Spissistilus festinus* is not a pest of wine grapes but an epidemiological vector of GRBV with virus acquisition and transmission occurring in northern California vineyards [8]. Transmission of GRBV by *S. festinus* is circulative, indicating that the transit of the virus through the salivary glands of *S. festinus* is a requisite for virus spread to occur [2]. Given this mode of transmission, testing whole bodies of *S. festinus* informs GRBV ingestion but not acquisition; it is only the testing of salivary glands that documents virus acquisition [9,10]. The transmission of GRBV by *S. festinus* is also non-propagative, meaning the virus does not use this treehopper as a host for its replication [2]. 

Geminiviruses such as GRBV are transmitted by distinct arthropod vectors in a virus genus-specific manner [9]. Therefore, it is plausible that other treehoppers may act as vectors of GRBV [10,11]. Several Membracidae species from the Ceresini tribe that consists of buffalo treehoppers and allies are present in North American vineyard ecosystems, including *Stictocephala* spp. and *Tortistilus* spp. [12,13,14,15,16]. Recently, *Stictocephala bisonia* and *Stictocephala basalis* were reported as potential vectors of GRBV [14]. However, the transmission assays used in this study relied on an artificial sucrose solution rather than plant material, thus precluding a definite conclusion on the role of these two *Stictocephala* species in the transmission of GRBV. Therefore, more work is needed to ascertain the role of *Stictocephala* spp. as vectors of GRBV [10]. 

No information is available on the potential role of *Tortistilus* spp. as vectors of GRBV. Given that *S. festinus* is a proven vector of GRBV [2,3,8], and the relatedness of the genera *Spissistilus* and *Tortistilus* within the Ceresini tribe [17], it is reasonable to speculate that *Tortistilus* spp. could be vectors of GRBV. Identifying a new vector of a grapevine virus, including GRBV, can be technically challenging [10]. An elegant approach to circumvent some of the inherent limitations related to the *Vitis*–GRBV pathosystem consists of testing the presence of GRBV in the salivary glands of vector candidates, for example, *Tortistilus* spp., that are caught in red-blotch-diseased vineyards [10]. If the virus is confirmed in the salivary glands of vector candidates, performing transmission assays would be a logical next step to ascertain their role as vectors of GRBV [10]. In contrary, if the virus is not detected in the salivary glands of vector candidates, performing follow-up transmission assays is not justified [10].

To address the potential role of *Tortistilus* spp. in the transmission of GRBV, vineyard populations of *Tortistilus* were surveyed in northern California during three consecutive growing seasons. Here, we summarize our efforts to characterize the nature of the *Tortistilus* spp. specimens caught on yellow panel cards in 102 vineyard sites and by sweep netting in proximal vegetation, and evaluate their ability to ingest and acquire GRBV.

## 2. Materials and Methods

### 2.1. Tortistilus and Spissistilus Collections

Adult specimens of treehoppers from the Ceresini tribe were collected in 102 vineyard sites in Napa Valley in California, USA, using yellow panel traps (6–8 traps per site) from June through November in 2021, and March through November in 2022 and 2023. Additional yellow panel cards were placed at the vineyard’s edge and in riparian habitats. Panel traps were rotated biweekly. The *Tortistilus* collections from traps were supplemented with specimens collected by sweep netting in vegetation proximal to vineyards (Table 1). *Spissistilus festinus* specimens were caught in the same vineyard sites in Napa Valley, California, as *Tortistilus* specimens and solely used in this study for GRBV ingestion and acquisition comparisons.

**Figure 1:**
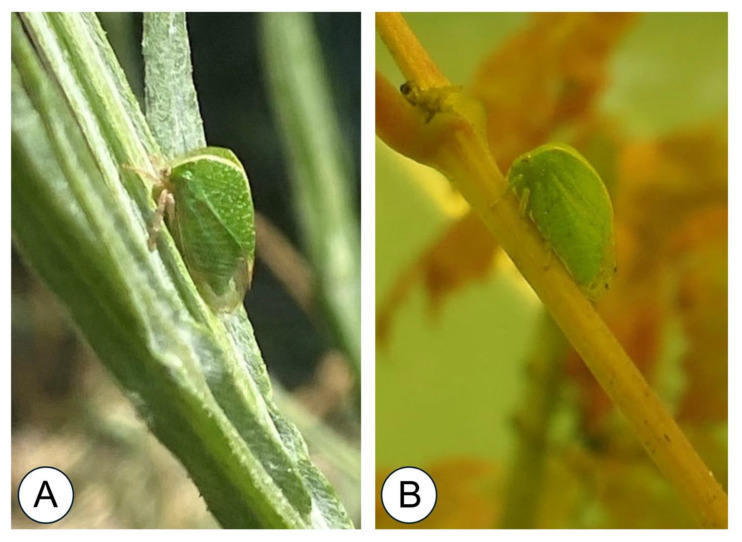
Depiction of a *Tortistilus wickhami* (**A**) and a *Spissistilus festinus* (**B**) with a differential morphological shape. Note the pronotum rising vertically above the head with lateral ridges joining over the thorax for *T. wickhami*, compared with the pronotum gradually curving backwards for *S. festinus.*

Specimens of *Tortistilus* were identified based on external morphological characteristics such as a large body size (5–8 mm), a pronotum rising vertically above the head with lateral ridges joining over the thorax, and, eventually, lateral pronotal horns. These morphological characteristics were clearly distinct from those of *S. festinus* specimens, which have a smaller body size (4–6 mm), a pronotum gradually curving backwards with lateral ridges joining midway to the length of the body, and no lateral horns (Figure 1). Whole *Tortistilus* and *S. festinus* insects were removed from panel cards using Goo Gone and jeweler’s forceps and visually categorized by morphology and sex. Then, specimens were stored at −80 °C in 2 mL Eppendorf tubes containing two sterile ball bearings for GRBV testing. 

### 2.2. Nucleic Acid Extraction and GRBV Testing Using PCR

Total DNA was extracted from individual *Tortistilus* and *S. festinus* using the MagMAX^TM^ DNA Multi-Sample Ultra Kit (ThermoFisher Scientific, Waltham, MA, USA) on a KingFisher^TM^ instrument. *Tortistilus* and *S. festinus* specimens were tested for the presence of GRBV by multiplex PCR using primer pairs targeting the coat protein (CP) and replicase protein (repA), as previously described [2]. Individual *Tortistilus* and *S. festinus* were dissected into heads with salivary glands and guts prior to DNA extraction and GRBV testing by multiplex PCR and qPCR for the assessment of GRBV acquisition [2,10,18].

### 2.3. Sequencing of Tortistilus ITS and mt-COI DNA Fragments 

PCR products targeting the mitochondrial cytochrome C oxidase 1 (mt-COI) gene and the nuclear internal transcribed spacer 2 (ITS2) region were obtained from individual *Tortistilus* using total DNA and universal primers designed in the mt-COI gene (LCO1490 and HCO1298) and the ITS2 region (Cas5p8Fc and Cas28b1d) [19,20,21]. PCR products were resolved by electrophoresis on 1% agarose gels, stained using GelRED^®^ (Biotium, Fremont, CA, USA), and visualized under ultraviolet (UV) illumination. PCR products were purified using the DNA Clean & Concentrator-5 PCR Purification Kit (Zymo Research Corporation, Irvine, CA, USA) and Sanger-sequenced at the Cornell University Biotechnology Resource Center (Ithaca, NY, USA).

### 2.4. Sequence Analyses and Phylogenetic Relationships

Sequences of the mt-COI and ITS2 fragments were analyzed using the Lasergene software suite and aligned in MegAlign Pro (Version 17.2.1) using MUSCLE [21,22]. Phylogenies were constructed using RaxML [21,23]. Branching confidence was estimated from 1000 bootstrap replicates for Maximum Likelihood analyses. Based on the overall quality of sequences, both ends were manually edited, and 569 nucleotides were considered in the analysis of mt-COI sequences and 381 nucleotides in the analysis of ITS2 sequences. Mitochondrial gene references of *Tortistilus wickhami* (KR576466, KR576085, MG513587), *Tortistilus inermis* (KF920404, KF920231), *Tortistilus pacificus* (KF919816, KF920297), *Tortistilus minutus* (KF920088, KF920333), *Spissistilus festinus* (MN888497-MN888502), *Stictocephala basalis* (MG510412, MG507627, KF920303), *Stictocephala bisonia* (PP708026, PP708027), *Stictocephala diceros* (KF919808, MG398647), and *Hadrophallus bubalus* (KF920331, KR575555) were retrieved from GenBank for sequence analyses. Since only references for *S. festinus* (MN887238-MN887243) ITS2 sequences are available in GenBank for Ceresini tribe treehoppers, specimens of *Stictocephala bisonia*, *Stictocephala diceros*, and *Hadrophallus bubalus* were collected from natural habitats in New York and sequenced for mt-COI and ITS2 fragments to strengthen our analyses.

### 2.5. Diagnostic PCR for Distinction between Tortistilus and Spissistilus Species

Morphologically, *Tortistilus* and *Spissistilus* can be difficult to distinguish. Therefore, diagnostic PCR assays were developed in the mt-COI gene with primers designed in this study for *Tortistilus* TWICKcoiF, 5’-TTCGAGTTGAACTAGGGC-3’, and TWICKcoiR, 5’-GGATATACTGTTCATCCCGTC-3’, and primers previously described for *S. festinus* TCAHcoiWestF, 5′-GAATTGGGACAACCAGGACC-3′, and TCAHcoiWestR, 5′-AACTGGAAGAGACATGAGG-3′ [21]. PCR reactions used 2.5 µL of 10x PCR buffer (Qiagen, Germantown, MD, USA), 1.0 µL of each primer at 10 µM, 0.25 µL of dNTP mix (10 mM each, deoxynucleotide triphosphate) (Qiagen), 0.125 µL of HotStar Taq polymerase (Qiagen), and 4.625 µL of nuclease-free water in a final volume of 12.5 µL [21]. Water was used as a negative control for the two types of PCR that were conducted using the following cycling protocol: 95 °C for 5 min; 30 cycles of 94 °C for 30 s, 56 °C for 60 s, 72 °C for 60 s; 72 °C for 10 min with *T. wickhami* primers and 95 °C for 5 min; 30 cycles of 94 °C for 30 s, 62 °C for 60 s, 72 °C for 60 s; and 72 °C for 10 min with *S. festinus* primers to amplify 314 bp and 496 bp DNA products, respectively [21]. Amplicons were resolved by gel electrophoresis on 1.5% agarose gels, stained with GelRED^®^ (Biotium) and imaged under UV light.

## 3. Results

### 3.1. Vineyard Sites and Tortistilus Specimen Collections

**Figure 2:**
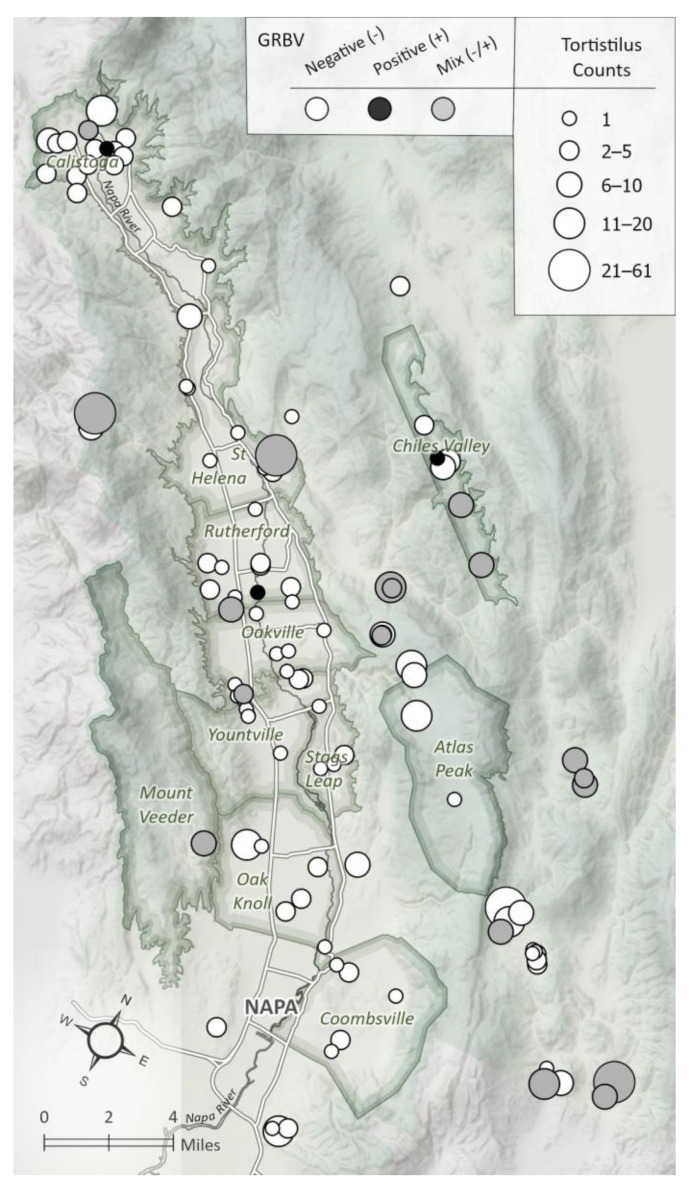
Location of the 102 vineyard sites selected for this study in Napa Valley, California, USA, depicting the abundance of *Tortistilus* and the presence of grapevine red blotch virus (GRBV) in specimens collected, as shown by PCR. Positive (+) indicates only GRBV positive specimens; Mix (-/+) indicates a combination of GRBV positive and negative specimens; and Negative (-) indicates only GRBV negative specimens at each of the 102 vineyard sites.

Our study was carried out over three growing seasons, across 102 vineyard sites in Napa Valley, CA, including areas in Saint Helena, Carneros, Calistoga, Rutherford, Oakville, Yountville, Chiles Valley, Mount Veeder, Oak Knoll, Coombsville, and Atlas Peak (Figure 2). Vineyard compositions included a mix of nine cultivars (Cabernet Sauvignon, Cabernet franc, Merlot, Sauvignon blanc, Chardonnay, Malbec, Petit Verdot, Petite Sirah, Zinfandel) and eight rootstock genotypes (110R, 101-14, 3309C, St George, 1103P, 1616C, 420A, 039-16). Vineyard sites varied by age (ranging from 4 to 27 years), low to high red blotch disease pressure (ranging from 1% to 78%), and a range of cover crop mixtures.

**Figure 3:**
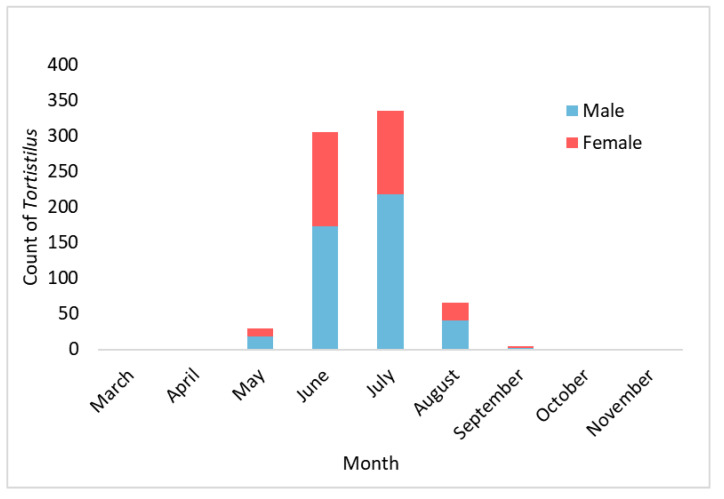
The cumulative distribution of *Tortistilus* collected by sex, in northern California vineyards, over three growing seasons (June to November in 2021 and March to November in 2022 and 2023).

From June through November in 2021 and March through November in 2022, a total of 758 *Tortistilus* specimens were caught in 102 vineyard sites using yellow panel traps or sweep netting captures, including 444 males and 294 females. Twenty specimens could not be sexed due to physical damage during removal from panel traps. Morphotyping for the presence or absence of horns revealed far more unhorned (82%, 618/758) than horned (18%, 140/758) *Tortistilus* specimens. The earliest caught *Tortistilus* was on 7 March 2022 and the latest was on 2 November 2022. Overall, the peak presence of *Tortistilus* occurred in June and July (Figure 3), as previously observed for *S. festinus* [5,24,25]. In total, 581 *Tortistilus* specimens were derived from panel traps, with 528 specimens captured on traps placed at the vineyard edge and 53 specimens captured on traps placed within vineyard rows. The remaining 177 were collected using sweep nets or captured by hand upon sight. 

### 3.2. Morphological Variability of Tortistilus Populations from Northern California Vineyards

**Figure 4:**
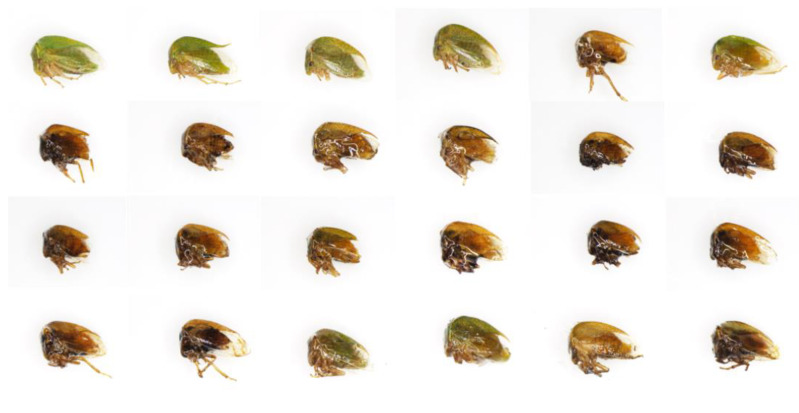
A side angle view of 24 *Tortistilus wickhami* specimens collected in northern California vineyards obtained under a SZX16 stereoscope (Olympus, Center Valley, PA, USA). Photographs were captured using the cellSense Standard software (version 1.18).

**Figure 5:**
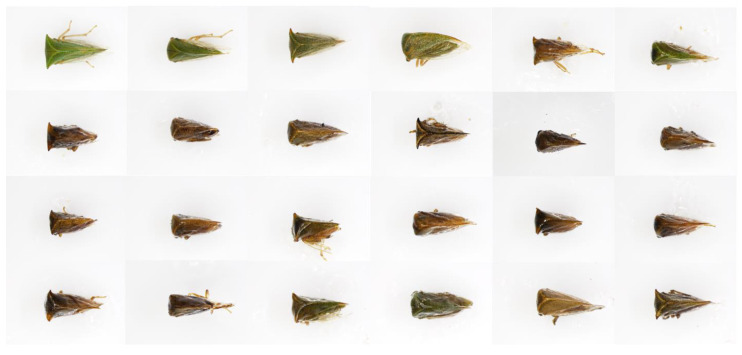
A dorsal view of the same 24 *Tortistilus wickhami* specimens shown in Figure 4 obtained under a SZX16 stereoscope (Olympus, Center Valley, PA, USA). Photographs were captured using the cellSense Standard software (version 1.18).

**Figure 6:**
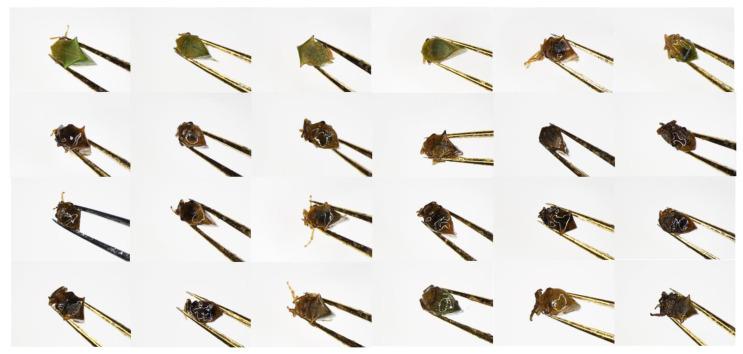
A face view of the same 24 *Tortistilus wickhami* specimens shown in Figure 4 obtained under a SZX16 stereoscope (Olympus, Center Valley, PA, USA). Photographs were captured using the cellSense Standard software (version 1.18).

A large degree of morphological variability was observed among *Tortistilus* specimens. To capture the diversity across varying sites of origin, sex, and morphotype, a representative set of 24 specimens was observed under a SZX16 stereoscope (Olympus, Center Valley, PA, USA) and photographed using the cellSense Standard software (version 1.18), capturing a side angle view (Figure 4), dorsal view (Figure 5), and face view (Figure 6) of each specimen. The average size of *Tortistilus* specimens ranged from 4.0 mm to 5.60 mm, with females tending to be larger than males. Two morphotypes were observed with some specimens having pronotal horns, and others were unhorned. A wide range of variation was observed for the length of the pronotal horns, with the shortest length being 0.25 mm on each side of the pronotum and 0.69 mm being the longest. The average length for pronotal horns was 0.47 mm (Figure 4, Figure 5 and Figure 6) (Supplementary Table S1).

### 3.3. Taxonomic Identification of Tortistilus Populations from Northern California Vineyards

**Figure 7:**
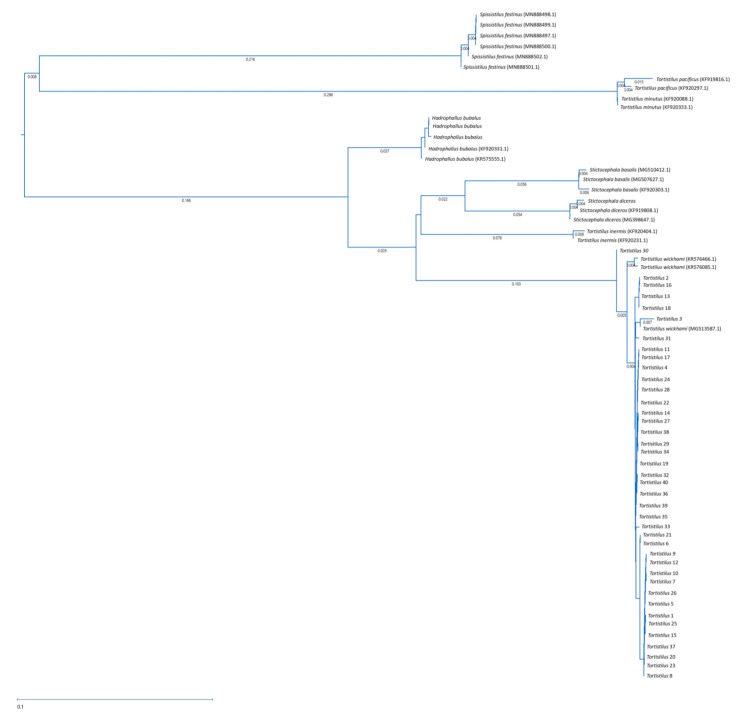
Phylogeny of partial mitochondrial cytochrome C oxidase I (mt-COI) sequences from *Tortistilus* populations collected from various sites and years in northern California vineyards produced by the Maximum Likelihood analysis with 1000 bootstrap replicates. Sequences derived from specimens collected in this study are listed without accession numbers and correspond to the information in Table 1, while the remaining sequences were retrieved from GenBank.

Sequences of mt-COI (569 bp) and ITS2 (381 bp) fragments of 7–21 *Tortistilus* specimens from each collection year, multiple vineyard sites, both morphotypes, and both sexes were obtained (Table 1). The within-*Tortistilus* population nucleotide sequence variation for the mt-COI sequence ranged from 0 to 2% and was up to 28% with other Ceresini treehoppers. Phylogenetic analysis of mt-COI sequences revealed that all 40 *Tortistilus* specimens grouped monophyletically with *T. wickhami*, apart from additional Ceresini tribe treehoppers for which sequences were retrieved from GenBank (Figure 7).

**Figure 8:**
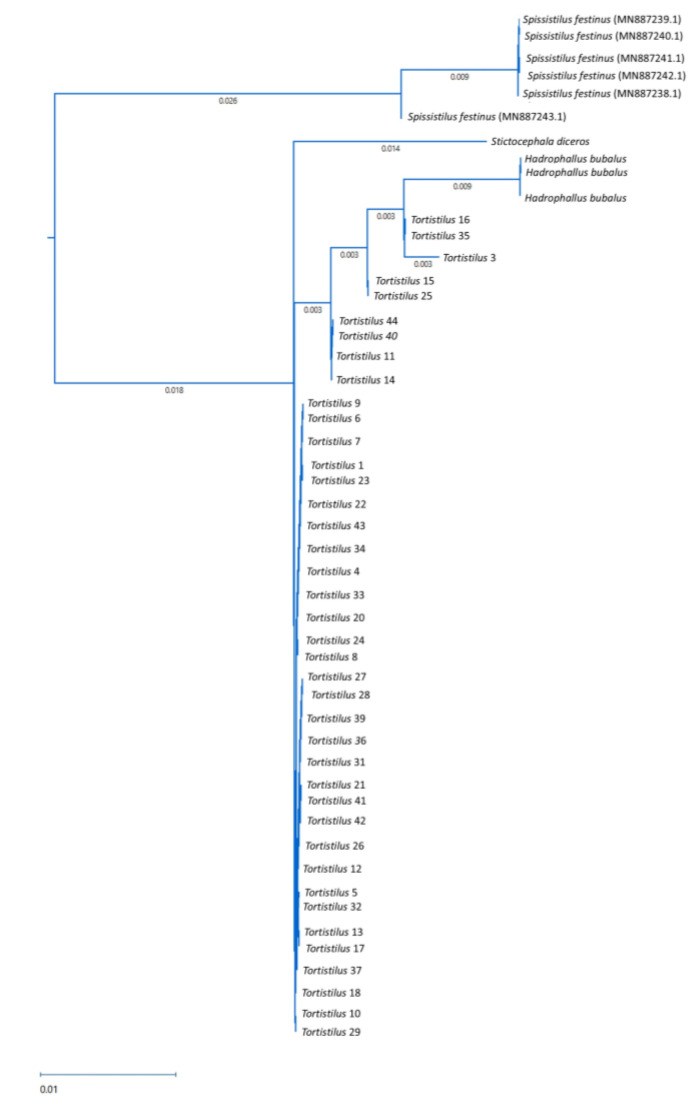
Phylogenetic analysis of partial internal transcribed spacer 2 (ITS2) sequences from *Tortistilus* populations collected from various sites and years in northern California vineyards produced by Maximum Likelihood analysis with 1000 bootstrap replicates. *Tortistilus* sequences are derived from specimens listed in Table 1. Additional sequences are from Ceresini treehoppers sourced from New York or retrieved from GenBank.

The within-*Tortistilus* population nucleotide sequence variation for the ITS2 sequence varied from 0 to 1% and was up to 4% with the sequence of other Ceresini treehoppers, including *S. festinus*. Phylogenetic analysis indicated a monophyletic grouping of *Tortistilus*, separate from other Ceresini treehoppers, including *S. festinus* from northern California vineyards and *Hadrophallus bubalus* and *Stictocephala diceros* from natural habitats in New York (Figure 8). No ITS2 sequences from other Ceresini treehoppers, including *T. wickhami*, are available in GenBank. Therefore, the *Tortistilus* specimens from California vineyards could not be definitively identified at the species level based on ITS2 sequences, except that they were distinct from *S. festinus* from California and *Stictocephala diceros* and *Hadrophallus bubalus* from New York. However, based on the monophyletic grouping observed with the partial ITS2 and mt-COI sequences, and the identification at the species level specimens based on partial mt-COI sequences, it was reasonable to assume that *Tortistilus* specimens from northern California vineyards corresponded to *T. wickhami.*

### 3.4. GRBV Acquisition by Tortistilus wickhami from Northern California Vineyards

Of the 758 *T. wickhami* specimens caught in 2021–2023, only 6% (48/758) tested positive for GRBV in multiplex PCR when analyzing whole bodies, including 6% (28/444) of males and 7% (20/294) of females. Interestingly, slightly more horned (9%, 12/140) than unhorned (6%, 36/618) specimens tested positive for GRBV, indicating ingestion of the virus. By comparison, 23% (59/259) of *S. festinus* collected from the same vineyard sites in Napa Valley, California, tested positive for GRBV in multiplex PCR in 2021–2022, with more males (17%, 22/132) than females (8%, 3/37) testing positive for GRBV in 2022 [26]. Furthermore, none (0/50) of the dissected heads with salivary glands of a small subset of *T. wickhami* tested positive for GRBV in multiplex PCR, while 6% (3/50) of the guts of the dissected specimens tested positive for GRBV. These results were confirmed by qPCR with identical Ct values (35–40) for the *T. wickhami* heads with salivary glands and for colony reared *S. festinus* that were used as a negative control (36), illustrating no virus acquisition. By comparison, 52% (12/23) of dissected heads with salivary glands and 65% (15/23) of dissected guts from *S. festinus*, a known vector of GRBV, that were collected at these same vineyard sites tested positive for GRBV in PCR. This result revealed a lack of virus acquisition by *T. wickhami*. 

### 3.5. Diagnostic Polymerase Chain Reaction for Tortistilus wickhami

**Figure 9:**
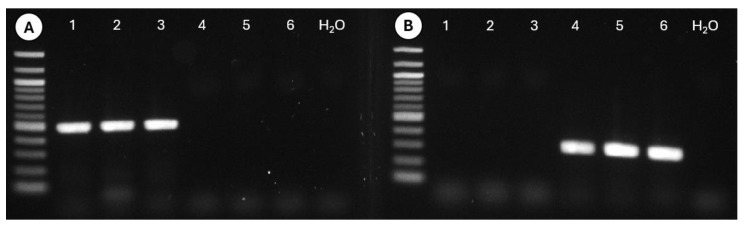
Diagnostic polymerase chain reaction for DNA-sequence-based identification of *Spissistilus festinus* (lanes 1–3, 496 bp) and *Tortistilus wickhami* (lanes 4–6, 314 bp) specimens from northern California using *S. festinus* TCAHcoiWestF and TCAHcoiWestR primers (**A**) or *T. wickhami* TWICKcoiF and TWICKcoiR primers (**B**). Lane 6 is a water control.

A PCR assay was designed to identify vineyard populations of *T. wickhami* or *S. festinus* from northern California by amplifying a 314 bp or 496 bp fragment of the mt-COI gene, respectively. The sensitivity of the assay was determined through the specific amplification of DNA targets only from the *S. festinus* (Figure 9A) or *T. wickhami* (Figure 9B) specimens with specific primer pairs. These results validated the use of both primer pairs in PCR for diagnostic assessment of *T. wickhami* and *S. festinus* from northern California vineyards. 

## 4. Discussion

*Spissistilus festinus* is only the second documented treehopper involved in the transmission of a plant virus. The first example is *Micrutalis malleifera*, which transmits tomato pseudo-curly top virus (TPCTV, genus *Topocuvirus*, family *Geminiviridae*) [27]. The spatiotemporal spread patterns of TPCTV in Florida tomato fields suggested minimal secondary spread and decreasing rates of infection as the distance from the field margin increased [28]. Similar epidemiological trends have been found for GRBV in some diseased vineyards in northern California [2,3,5,8]. 

Given that *S. festinus* is a known vector of GRBV [2,3,8], *Tortistilus* spp. were hypothesized to be vector candidates of GRBV due to the close relatedness of these two treehopper species [17]. *Tortistilus* can be found throughout the Pacific northwest, with *Tortistilus wickhami* being the most prevalent in northern California and *Tortistilus pacificus* in southern California [29]. Based on their presence in vineyards of northern California, it was important to ascertain the potential role of *Tortistilus* as a vector of GRBV in northern California vineyards. Here, we documented that *Tortistilus* specimens caught in 102 vineyard sites in northern California during three consecutive years correspond to *T. wickhami* based on mt-COI barcode sequences. None of the *T. wickhami* caught in vineyards, including in some red blotch diseased vineyards, acquired GRBV. Indeed, none of the heads with salivary glands of *T. wickhami* (0%, 0/50) tested positive for GRBV in multiple PCR and qPCR. In contrast, approximately half of the dissected heads with salivary glands of *S. festinus* (52%, 12/23) from the same collection vineyards as *T. wickhami* tested positive for GRBV in multiple PCR. Therefore, the role of *T. wickhami* as a vector of GRBV is doubtful, and there is no strong justification to perform follow-up virus transmission assays [10]. 

More *T. wickhami* specimens (*n* = 758) were collected in 2021–2023 (this study) than *S. festinus* specimens (*n* = 259) in 2021–2022 [26] in Napa Valley, CA. As has been observed for *S. festinus*, the peak observations of *T. wickhami* in northern California vineyard ecosystems were in June and July, though these peaks were far more finite with limited visibility outside this window of time. Unlike *S. festinus,* which are not readily trapped via yellow panel cards or sticky traps [24,26,30,31], the majority of *T. wickhami* collected in this study were derived from passive traps (*n* = 581) in the vineyard ecosystems rather than more active methods such as sweep netting (*n* = 177). This suggests that *T. wickhami* relies on vineyard ecosystems for feeding during the summer in northern California. While collecting *Tortistilus* specimens for this study, we observed that they were often clustered together, for example, on yellow star thistles, *Centaurea solstitialis*, at the vineyard edge. *Tortistilus* specimens derived from the yellow panel traps were almost exclusively intercepted at the vineyard edge (*n* = 528) rather than internally placed within vineyard rows (*n* = 53). Group-living behaviors could explain the trends observed for *T. wickhami*, including increased numbers of specimens collected at the edge of vineyards and an isolated seasonal peak in vineyard ecosystems. It is also possible that weather patterns might influence the dispersal of *T. wickhami* in vineyard ecosystems. More work is needed to address these issues. Further analysis of *T. wickhami* distribution and movement in Napa Valley, CA, in comparison with *S. festinus*, could assist in the monitoring of these cryptic species, which seem to behave quite differently in these systems.

Moreover, a reduced proportion of *T. wickhami* (6%, 48/758) tested positive for GRBV in 2021–2023 (this study) in comparison with *S. festinus* (23%, 59/259) in 2021–2022 [26], revealing a lower virus ingestion rate. Furthermore, an equal proportion of *T. wickhami* females (7%, 20/294) and males (6% (28/444) tested positive for GRBV in 2021–2023 (this study), while more *S. festinus* males (17%, 22/132) than females (8%, 3/37) from the same vineyard sites tested positive for GRBV in 2022 [26]. A lack of difference in GRBV ingestion by sex for *T. wickhami* might suggest distinct behavior in vineyards compared with *S. festinus*. 

To facilitate the study of treehoppers as vectors of GRBV, it is important to improve the identification of species beyond morphological traits. Previous morphological classification of *Tortistilus* specimens separated species into genera based on the presence or absence of horns. For example, horned *Tortistilus* specimens from California were considered *T. albidosparsus*, and unhorned *Tortistilus* specimens were considered *T. wickhami* [32]. This classification was later found to be inaccurate, as confirmed in this study, and specimens began to be reassigned into genera based on genitalic traits [29,32,33]. In 1979, the Ceresini tribe was revisited and revised to the generic level [34] with many new and synonymous genera observed but only minor changes made to the tribe [35,36,37,38,39]. With the advancement of genetic barcoding and phylogenetic analyses, members of the Ceresini tribe have been demonstrated to be monophyletic, sharing one common ancestor [17,40,41], but this assessment has not been used to understand the relationships and taxonomic classifications of the genera within the tribe. Additional phylogenetic analyses may help to clarify relationships between members of the Ceresini tribe as well as synonymies. *T. wickhami* and *S. festinus* appear cryptic to an untrained investigator. For this reason, molecular diagnostic tools are important for facilitating the accurate identification of the treehopper species present in vineyard sites, particularly those where secondary spread of GRBV is observed. To this end, the *T. wickhami* diagnostic PCR developed in this study should be helpful. Unfortunately, the *T. wickhami* primers cannot be utilized in a multiplex PCR with the *S. festinus* primers due to differences in annealing temperatures and similarities in amplicon size. To maximize the efficiency of diagnostic testing of treehoppers from vineyard ecosystems in northern California, further optimization of these assays should be prioritized.

Beyond *S. festinus* and *T. wickhami*, additional treehopper species have been identified as vector candidates of GRBV, including *Stictocephala basalis* and *Stictocephala bisonia*, both members of the Ceresini tribe, as well as *Entylia carinata* and *Enchenopa binotata* [14,15], all well distributed throughout North America. However, the transmission capacities of these species remain to be affirmed [10]. The study of treehoppers as vectors of viruses, including GRBV, is in its infancy. Additional vectors of GRBV are likely to be identified, although further work is needed to increase our understanding of the relatedness of treehoppers in relation to their vector candidacy.

## Figures and Tables

**Table 1:** Information on the 44 *Tortistilus wickhami* specimens and a few *Hadrophallus* and *Stictocephala* pecimens characterized in this study including their location of origin, year of collection, morphotype, sex, and GenBank accession number of partial mt-COI or ITS2 sequences.

Name	Location	Year	Morphology	Sex	GenBank Accession Number	
					mt-COI	ITS2
*Tortistilus* 1	Rutherford, CA, USA	2023	Unhorned	Female	PQ096090	PQ108530
*Tortistilus* 2	Rutherford, CA, USA	2023	Horned	Female	PQ096091	-
*Tortistilus* 3	Oak Knoll District, CA, USA	2023	Horned	Male	PQ096092	PQ108531
*Tortistilus* 4	Oak Knoll District, CA, USA	2023	Unhorned	Female	PQ096093	PQ108532
*Tortistilus* 5	Oak Knoll District, CA, USA	2023	Unhorned	Female	PQ096094	PQ108533
*Tortistilus* 6	Oak Knoll District, CA, USA	2023	Horned	Female	PQ096095	PQ108534
*Tortistilus* 7	Chiles Valley, CA, USA	2023	Unhorned	Male	PQ096096	PQ108535
*Tortistilus* 8	Chiles Valley, CA, USA	2023	Unhorned	Male	PQ096097	PQ108536
*Tortistilus* 9	Chiles Valley, CA, USA	2023	Horned	Male	PQ096098	PQ108537
*Tortistilus* 10	Chiles Valley, CA, USA	2023	Unhorned	Female	PQ096099	PQ108538
*Tortistilus* 11	Chiles Valley, CA, USA	2023	Horned	Male	PQ096100	PQ108539
*Tortistilus* 12	Chiles Valley, CA, USA	2023	Unhorned	Male	PQ096101	PQ108540
*Tortistilus* 13	Chiles Valley, CA, USA	2023	Horned	Male	PQ096102	PQ108541
*Tortistilus* 14	Chiles Valley, CA, USA	2023	Unhorned	Male	PQ096103	PQ108542
*Tortistilus* 15	Chiles Valley, CA, USA	2023	Unhorned	Male	PQ096104	PQ108543
*Tortistilus* 16	Chiles Valley, CA, USA	2023	Unhorned	Male	PQ096105	PQ108544
*Tortistilus* 17	Calistoga, CA, USA	2023	Unhorned	Female	PQ096106	PQ108545
*Tortistilus* 18	Calistoga, CA, USA	2023	Horned	Female	PQ096107	PQ108546
*Tortistilus* 19	Spring Mtn. District, CA, USA	2023	Unhorned	Female	PQ096108	-
*Tortistilus* 20	Spring Mtn. District, CA, USA	2023	Horned	Male	PQ096070	PQ108547
*Tortistilus* 21	Napa, CA, USA	2022	Horned	Male	PQ096071	PQ108548
*Tortistilus* 22	Napa, CA, USA	2022	Unhorned	Male	PQ096072	PQ108549
*Tortistilus* 23	Calistoga, CA, USA	2022	Horned	Male	PQ096073	PQ108551
*Tortistilus* 24	St. Helena, CA, USA	2022	Unhorned	Female	PQ096074	PQ108552
*Tortistilus* 25	Yountville, CA, USA	2022	Horned	Male	PQ096075	PQ108553
*Tortistilus* 26	Gordon Valley, CA, USA	2022	Unhorned	Male	PQ096076	PQ108554
*Tortistilus* 27	St. Helena, CA, USA	2022	Horned	Female	PQ096077	PQ108556
*Tortistilus* 28	Coombsville, CA, USA	2021	Unhorned	Male	PQ096078	PQ108557
*Tortistilus* 29	Gordon Valley, CA, USA	2021	Unhorned	Male	PQ096079	PQ108558
*Tortistilus* 30	Oak Knoll District, CA, USA	2021	Unhorned	Female	PQ096080	-
*Tortistilus* 31	Pope Valley, CA, USA	2021	Unhorned	Female	PQ096109	PQ108559
*Tortistilus* 32	Oak Knoll District, CA, USA	2021	Unhorned	Male	PQ096081	PQ108560
*Tortistilus* 33	Pope Valley, CA, USA	2021	Unhorned	Male	PQ096082	PQ108561
*Tortistilus* 34	Wooden Valley, CA, USA	2021	Unhorned	Female	PQ096083	PQ108562
*Tortistilus* 35	Pope Valley, CA, USA	2022	Unhorned	Female	PQ096084	PQ108563
*Tortistilus* 36	Pope Valley, CA, USA	2022	Unhorned	Male	PQ096085	PQ108564
*Tortistilus* 37	Pope Valley, CA, USA	2022	Unhorned	Male	PQ096086	PQ108565
*Tortistilus* 38	Pope Valley, CA, USA	2022	Unhorned	Male	PQ096087	-
*Tortistilus* 39	Pope Valley, CA, USA	2022	Unhorned	Female	PQ096088	PQ108566
*Tortistilus* 40	Pope Valley, CA, USA	2022	Unhorned	Female	PQ096089	PQ108567
*Tortistilus* 41	Chiles Valley, CA, USA	2023	Horned	Female	-	PQ108568
*Tortistilus* 42	Yountville, CA, USA	2022	Horned	Male	-	PQ108550
*Tortistilus* 43	St. Helena, CA, USA	2022	Unhorned	Female	-	PQ108555
*Tortistilus* 44	Pope Valley, CA, USA	2022	Unhorned	Female	-	PQ108569
*Hadrophallus bubalus*	Geneva, NY, USA	2022	Horned	Female	PQ096110	PQ108570
*Hadrophallus bubalus*	Geneva, NY, USA	2022	Horned	Male	PQ096111	PQ108571
*Hadrophallus bubalus*	Geneva, NY, USA	2022	Horned	Female	PQ096112	PQ108572
*Stictocephala diceros*	Lyons, NY, USA	2023	Horned	Female	PQ096113	-
*Stictocephala diceros*	Lyons, NY, USA	2023	Horned	Male	-	PQ108573

## Data Availability

Raw data will be made available upon request. Voucher specimens of *Tortistilus wickhami* and *Spissistilus festinus* from northern California vineyards are available upon request.

## Supplementary Materials

The following supporting information can be downloaded at https://www.mdpi.com/article/10.3390/insects15090664/s1: Table S1: Measurements of morphological traits of 24 *Tortistilus wickhami* based on photos captured under a SZX16 stereoscope (Olympus, Center Valley, PA, USA) and using the cellSense Standard software (version 1.18).
