# Supplementary material for: Assessing the Potential of Tortistilus (Hemiptera: Membracidae) from Northern California Vineyards as Vector Candidates of Grapevine Red Blotch Virus
Source: Insects. 2024 Aug 31;15(9):664. doi: 10.3390/insects15090664 (PMC11432720; doi:10.3390/insects15090664)
Supplement: Supplementary file 1 [file insects-15-00664-s001.zip › insects-3158759-supplementary.pdf]

Supplementary Table S1. Measurements of morphological traits of 24 *Tortistilus wickhami* based on photographs captured under a SZX16 stereoscope (Olympus, Center Valley, PA, U.S.A.) and using the cellSense Standard software (version 1.18). Body length was measured as a combined head, thorax, and abdomen from a side view. Body width was measured from socket (right side) to socket (left side) from an aerial view. Body height was measuring from mouth to pronotum from a face-up view. Horn length was measured from a face-up view for each of the two horns, and the final value was obtained by averaging the two values.

Specimen No.	Sex	Morphology	Length (mm)	Width (mm)	Height (mm)	Horns (mm)
1	Female	Horned	5.60	2.40	3.40	0.69
2	Female	Unhorned	5.20	2.20	3.20	0
3	Female	Horned	5.15	2.30	3.20	0.56
4	Female	Unhorned	5.40	2.20	3.10	0
5	Male	Horned	4.10	2.10	3.10	0.47
6	Female	Unhorned	5.00	2.20	2.80	0
7	Male	Horned	4.10	2.10	3.15	0.38
8	Male	Unhorned	4.40	2.10	3.00	0
9	Female	Unhorned	5.20	2.40	3.25	0
10	Male	Horned	4.90	2.30	3.00	0.50
11	Male	Unhorned	4.00	2.00	3.00	0
12	Male	Unhorned	4.80	2.10	2.60	0
13	Male	Horned	3.80	2.00	2.80	0.25
14	Male	Unhorned	4.40	2.20	2.70	0
15	Male	Horned	4.40	2.10	2.60	0.41
16	Female	Unhorned	5.30	2.15	2.90	0
17	Male	Horned	4.20	2.00	3.00	0.34
18	Male	Unhorned	4.20	2.15	3.20	0
19	Male	Horned	5.00	2.20	3.40	0.38
20	Male	Unhorned	4.20	2.05	2.70	0
21	Female	Horned	5.00	2.20	3.20	0.44
22	Female	Unhorned	5.40	2.50	3.60	0

23	Female	Unhorned	5.20	2.40	3.40	0
24	Male	Horned	4.40	2.20	3.20	0.50
